# Integrating Computational Thinking and Empowering Metacognitive Awareness in Stem Education

**DOI:** 10.3389/fpsyg.2022.872593

**Published:** 2022-06-09

**Authors:** Nagalaxmy Markandan, Kamisah Osman, Lilia Halim

**Affiliations:** Faculty of Education, National University of Malaysia, Bangi, Malaysia

**Keywords:** computational thinking, text-based programming, Biology, constructionism, metacognitive awareness, STEM education

## Abstract

Education digitization highly enthuses learners for deeper learning and developing thought processes in formulating problems and their solutions effectively in their real-life circumstances. Implementing computational thinking skills through programming in Malaysian primary and secondary school STEM curriculum create huge challenges, especially among STEM educators. This study highlights the integration of four major theories in developing the Metacognitive Empowerment by Computational Thinking (ME-CoT) learning module by cultivating computational thinking through programming skills to promote metacognitive awareness in Biology students. Pilot research was conducted to investigate the reliability of the ME-CoT learning module. Since the study sample was less than 30 students then, the consistency of the measurements, Pearson’s r was calculated to identify stability reliability. Findings revealed that the ME-CoT learning module has very strong stability reliability with a value of *r* = 0.974 and provides advantages such as assisting students to understand the content of the lesson more actively and in a fun way.

## Introduction

Education digitalization is having a significant impact on 21st-century learning, proving the conceptual underpinning of integrating technology in education 4.0 (Maharani et al., 2019; Karimah et al., 2020; Samri et al., 2020) to solve problems effectively and efficiently with broad applicability by employing computational thinking (Tsarava et al., 2019). Computational thinking is a set of problem-solving abilities that today’s learners must master and improve (Román-González et al., 2017), and it has progressively grown in importance as a means of thinking about addressing complicated or open-ended situations. Additionally, computational thinking equipped pupils with the ability to think critically, rationally, and systematically (Goyal et al., 2016; Susan and Nurfaradilla, 2019; Karimah et al., 2020, 2021; Samri et al., 2020) as well as to be a lifelong learner (Mohd Noor, 2012). Perhaps the most startling truth is that, because of its ability to debug or solve problems, computational thinking is always related to the metacognitive or cognitive domain. Facilitating problem-solving skills through programming skills will awaken students’ thinking skills and boost their metacognitive awareness. Even though many academics point out that computational thinking is not the same as programming, several studies have demonstrated that procedural and creative programming abilities can directly increase students’ cognitive and metacognitive strategies (Román-González et al., 2017).

According to research, computational thinking is closely linked to cognitive concepts such as coding and programming (Kazimoglu et al., 2012; Arihasnida et al., 2017; Samri et al., 2020) both are cognitive processes. According to previous research, programming is an effective and strategic way to improve problem-solving skills in students (Tsarava et al., 2019). Furthermore, involving students in programming activities is synonymous with the development of computational thinking skills. Computational thinking skills are the foundation of a student’s ability to think critically (Samri et al., 2020). These abilities are well-organized depending on the activities and tactics employed by the pupils to solve certain difficulties (Kamisah, 2022). When it came to computational thinking skills, the researcher introduced several of them, all of them based on computer programming or computing principles. Those skills were handled as a set of thinking skills in the way computer scientists will think, and it is a fundamental talent that everyone in the world should acquire (Wing, 2006, 2011; Kalelioglu et al., 2016; Zapata-Caceres et al., 2020).

The inclusion of computational thinking and programming skills in primary and secondary school curriculum exemplifies the Malaysian educational system’s massive paradigm change. Furthermore, higher education has been identified as the location for implementing computational thinking. However, current paradigm shifts in STEM education, as well as the need for Education 4.0, have demonstrated that this fundamental skill is required from early childhood (Falloon, 2016; Papadakis and Kalogiannakis, 2019; Otterborn et al., 2020), through primary (Haslina et al., 2018; del Olmo et al., 2020), secondary (Gillott et al., 2020), and finally higher education (Kalelioglu et al., 2016; Román-González et al., 2017). In comparison to adults or teachers, pupils’ ability to acquire computational thinking was crafted within Vygotsky’s Zone Of Proximal Development (Kalelioglu et al., 2016; Kotsopoulos et al., 2017) and it is an advanced level (Puganesri and Puteh, 2019). As a result, Malaysia’s Ministry of Education (MOE) has forged ahead and included computational thinking in the Standard Based Primary School Curriculum (Revised 2017) and Standard Based Secondary School Curriculum (Revised 2017), particularly in STEM education. Computational thinking is introduced in the classroom through an interdisciplinary approach in parallel to content knowledge. When traditional teaching methods are still used to deliver Biology content knowledge (Çimer, 2012; Lay Ah Nam and Kamisah, 2017; Bergan-Roller et al., 2018), a difficult scenario arises.

As we know most of the students who choose STEM Biology subjects are students who choose careers in medicine. Anatomy and physiology are core subjects in medical and health science programs which are often challenging programs compared to other disciplines (Periya and Moro, 2019). Moreover, student achievement in these anatomy and physiology subjects is closely related to student achievement in biology subjects starting from upper secondary school (Anderton et al., 2016). Teachers are responsible for contextual science knowledge, adapting it to the needs and demands of students and the curriculum to ensure significant learning occurs (Piaget, 1972a; Reinoso Tapia et al., 2019). In addition, topic content enriched with diagrams, processes, structures as well as biological literacy or facts describing biological processes (Reinoso Tapia et al., 2019) requires an interesting form of teaching and learning and relates to daily life as well as the mind-challenging questions (Fazilah et al., 2016) to attract students to create an active learning environment (Mohamad Masrizan, 2019; Reinoso Tapia et al., 2019). However, active learning cannot be practiced if rote learning (Fazilah et al., 2016) is practiced in schools. Symbiosis with that, abstract and complex facts are usually presented to students using lecture methods (Çimer, 2012; Lay Ah Nam and Kamisah, 2017; Bergan-Roller et al., 2018), as a contributing factor to passive learning. This method was chosen based on the simple factor of managing the class as well as simple techniques for completing the syllabus (Fazilah et al., 2016). In addition to raising issues of lack of motivation and interest (Lay Ah Nam and Kamisah, 2017), memorization learning methods increased the consequences of declining achievement in Biology subjects due to students lacking exposure to the problem -based learning leading to difficulty in answering HOTS questions. The memorization effect also led to a decrease in the achievement of the problem-solving test results of the Program for International Student Assessment (PISA) 2012 (Fazilah et al., 2016) and Trends in International Mathematics and Science Study (TIMSS) (KPM, 2016, 2017) in Malaysia. Thereby the number of students involved in Biology education decreases drastically in Malaysia. In addition, the participation of students in STEM is very worrying which can be seen through the issue of difficulty in achieving Policy 60:40 (Science/technical: literature).

Developing students’ learning thinking processes has become a key challenge for teachers in the classroom, and it necessitates a methodical teaching strategy and instrument (Bergan-Roller et al., 2018). Due to a lack of appropriate guidance and modules to assist teachers and students in implementing computational thinking (Cheah, 2016; Lay Ah Nam and Kamisah, 2017; Puganesri and Puteh, 2019; Karimah et al., 2021), the researcher has the opportunity to develop a specific module to integrate computational thinking via programming skills. Computational thinking skills classification proposed by Kalelioglu et al. (2016) and Burbaite et al. (2018) has been applied in this research to develop a Metacognitive Empowerment by Computational Thinking Module (ME-CoT) in Biology Education.

In today’s digital world, students should be well prepared with problem-solving abilities (Arihasnida et al., 2017; Samri et al., 2020), as noted in the Framework of Computational Thinking as Problem Solving (Kalelioglu et al., 2016). Burbaite et al. (2018) also blended Model Revised Bloom’s Taxonomy. Table 1 illustrates the computational thinking skills used to develop the ME-CoT module. As a result, the researcher took advantage of a former chance to combine the computational thinking skills from two well-developed frameworks to create an outstanding module with problem-solving computational thinking skills. Both frameworks stress essential computational problem-solving abilities such as abstraction, decomposition, pattern recognition, algorithms, modeling, simulation, and debugging. Those skills are well-organized to encourage students’ metacognitive awareness whilst still accumulating problem-solving computational thinking. The table below illustrates computational thinking in the context of problem-solving. The ME-CoT module organizes each computational thinking skill according to the problem-solving sequence described in the Framework of Computational Thinking as a Problem-Solving Process (Kalelioglu et al., 2016). Despite focusing on computational thinking as a problem-solving skill, several studies have shown that programming skills can be used to teach computational thinking (Pedaste et al., 2015; Karimah et al., 2020). The interrelationship between programming and computational thinking demonstrates that programming and cognitive processes are inextricably linked (Samri et al., 2020; Karimah et al., 2021). According to the research, computational thinking is more closely linked to cognitive processes than to computing (Li et al., 2020).

**TABLE 1:**
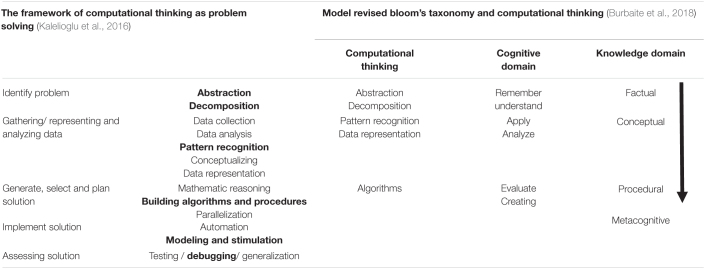
ME-CoT module’s computational thinking skills.

However, because the thinking process is directly tied to programming abilities, which are more prominent in computing, it has been demonstrated that incorporating programming skills will help to enhance computational thinking (Karimah et al., 2021). Several software systems have been developed to assist students in efficiently learning Biology by stimulating and modeling. Visual programming, commonly known as scratch, is used by primary school students to build their computing skills (Oluk and Korkmaz, 2016; Haslina et al., 2018). Even though various programming tools such as scratch, Microsoft Small Basic, Alice, and Toontalk have been introduced in primary school and focus on visual aspects such as drag and drop the blocks (Karimah et al., 2021), it is more valuable if the teacher introduces more advanced programming skills using programming languages for secondary school students to create a higher level of interest in learning programming and Biology content.

Furthermore, Rubinstein and Chor (2014) stated that rather than focusing on visual block programming such as Scratch, secondary students should be introduced to computational concepts and practices related to real programming languages such as Java, C+, C#, and C++, particularly in biology education (Rubinstein and Chor, 2014). This module is designed for novices who want to do some basic programming using the C# programming languages. As a result, having adequate models of computation systems (Aho, 2012) is critical to ensuring that students use the ME-CoT Visual Studio to develop their presentation or activity product. This Visual Studio Community 2019 was chosen because of its ability to focus on visuals or images, which is relevant to Biology education. In form four Biology lessons, the ME-CoT Module was implemented using computer programming using Visual Studio Software on the topic of Respiratory Systems in Humans and Animals. Numerous researchers have identified the necessity to examine programming tasks utilizing block-based programming such as scratch in the context of primary and secondary education from various STEM education fields. However, there are just a few studies that use text-based programming to test students’ computational thinking and metacognitive awareness skills. As a result, educational theories should be closely associated with the development and implementation of the Module using text-based programming. Thereby, the ME-CoT module was created based on the integration of three key learning theories: Cognitive Learning Theory, Social Learning Theory, and Constructivist Learning Theory, to measure students’ computational thinking skills and metacognitive awareness.

## Related Work

Creating a module in the realm of education that is closely tied to educational theory (Higgs, 2013). The research proposed constructionism theory to enable students actively participate in learning processes and make a product as a sign of learning outcome. After digging deep into educational theory, constructionism is proven to strengthen students’ computational thinking capacity and trigger metacognitive awareness. Rather than focusing on “learning by doing,” penetration of the constructionism theory in the ME-CoT Module was positively connected with “learning by making.” Students achieve a sufficient level of mastery over the topic knowledge and the general computational thinking talent, as well as operation of the metacognitive awareness in themselves, by making such a product.

Constructivist Theory, according to Piaget, indicates that students play a significant role and actively participate in the construction of their knowledge. Teachers serve simply as facilitators, not as knowledge builders, for their students. As a result, this research is founded on the constructivism idea, which states that students are responsible for the construction of existing knowledge. Additionally, the materials or products that students create as a result of programming demonstrate that pupils are capable of more effective learning. Papert also demonstrated as Piaget suggested, that learning occurs through the construction and reconstruction of knowledge through experience. Papert’s Constructionism, in particular, formulates learning in the context of a situation rather than looking at it from afar. Students’ involvement in current events can help them gain a better awareness of the lack of interaction with the environment (Ackermann, 2001).

Additionally, Vygotsky’s Social Theory of Constructivism is linked to both computational thinking and students’ metacognitive awareness. John Flavell, the first scientist to investigate the phenomenon of memory, cleared the path for other researchers to investigate the topic. A social interaction-specific metacognitive-related theoretical proposal (Flavell, 1979). The development of concepts available in pupils’ cognitive sets is aided by social contact. Cognitive development is linked to metacognitive development, which is the highest level of knowledge. Students’ cognitive development is aided by social contact, collaborative learning, and cooperative learning. Vygotsky’s Social Theory of Constructivism developed the notion of the ME-CoT Module, which provides a learning platform for students to connect with peers while completing tasks and implementing effective problem-solving skills in their respective Proximal Development Zones. Constructivism’s Social Theory strives to establish an understanding that gives significance to what is taught. According to the Social Theory of Constructivism, students build concepts through interaction until a new concept emerges, resulting in knowledge transformation among students in their respective Proximal Development Zones (ZPD). The Proximal Developmental Zone is defined as the distance between a child’s ability to perform a task under adult guidance and the child’s ability to solve a problem on his or her own (Vygotsky, 1979a). The authentic problem assigned in ME-CoT Module engages novice learners or students in text-based programming skills within a discipline or field of studies. Thereby, the text-based programming is doable at the appropriate level for the students in upper secondary. The module is also designed perfectly under Proximal Development Zones (ZPD), which is situated in the social context and involves active students’ participation as well as working as a community in a group.

The cognitive learning theory is essential since the implementation of each generated learning module takes place in the classroom, during teaching and learning activities to gain content knowledge in biology education. Cognitive learning theory explains that learning is a change - a change that occurs in the information available in a person’s memory. Cognitive learning theory is a new view to replace behaviorism theory that focuses on external stimuli. Moreover, the curiosity instinct that drives the mental process to understand and know a concept is fundamental to the theory of cognitive learning. Since this learning module is more focused on metacognitive which involves sensory memory and long-term memory, hence Robert Gagne’s Information Processing Theory is fundamental in the formation of students ‘metacognitive awareness. Gagne (1970) has sought a variety of superior and perfect ways to ensure learning takes place. According to Gagne (1970), a person receiving, and processing information received through the senses is like the information processing of a computer. Plans are inputs processed by sensory memory and short-term memory. The information generated will be stored in long-term memory or used to act with the environment. The programming activities (input) especially text-based programming applied through this module support the construction of students’ thinking through digital tools.

The metacognitive theory is closely related to Jean Piaget’s Theory of Cognitive Learning (1849–1936) and the social theory of Vygotsky’s Social Theory of Constructivism. Thinking about thinking is defined as metacognitive (Flavell, 1979; Sun, 2013; Mazli Sham and Saemah, 2014; Koc and Kuvac, 2016; Astuti et al., 2017). Thinking is a cognitive skill that entails mental activities that evolve by an individual’s ability to adapt at each level with the organization of the structure of thinking, which includes schema, assimilation, accommodation, and adaptation (Piaget, 1972b). Metacognitive is a person’s knowledge, control of thinking, and learning activities (Astuti et al., 2017; Chou, 2017). Effective learning will occur when students know what they know and what they need to know to fill the knowledge gap that exists where awareness exists within students. Metacognitive awareness refers to a person’s awareness of what they know and doesn’t know. Metacognitive strategies are methods that students employ to become more conscious of their thinking and learning processes. Students can use metacognitive awareness to help them govern their brains by planning, monitoring, and assessing what they’ve learned (Koc and Kuvac, 2016; Kyairaniah et al., 2017). Students are responsible for their knowledge. Metacognitive awareness is also very important in systematic problem solving as it is focused on computational thinking (Yağcı, 2019). Students’ metacognitive awareness will be automatically awakened by exposing them to computational thinking and text-based programming skills. Students will be aware of the importance of programming abilities in ensuring the correctness and well-designed performance of the final product. The ME-CoT Module focused on demonstrating problem-solving that might occur with input programming tasks, as well as running the program to find any possible errors.

**FIGURE 1:**
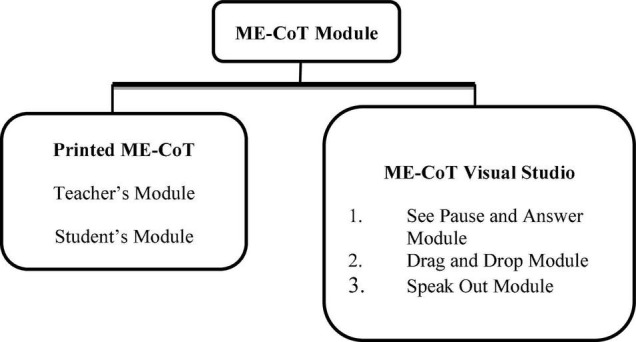
Component of ME-CoT module.

Since the module was created based on the four key theories, each activity was introduced in a staggered manner, beginning with the ME-CoT Visual Studio and continuing with the Printed Module The students then participated in a hands-on session in ME-CoT Visual Studio which was followed by an authentic activity. Figure 1 depicts the ME-CoT Module’s two major components.

## Module Development

The theoretical integration is capable of developing the interdisciplinary approach to STEM Education and computer science which will be implemented in the teaching and learning process in the classroom. This theoretical integration cladding the principal interaction of three major learning theories namely Cognitive Learning Theory, Social Learning Theory, and Constructivist Learning Theory.

**FIGURE 2:**
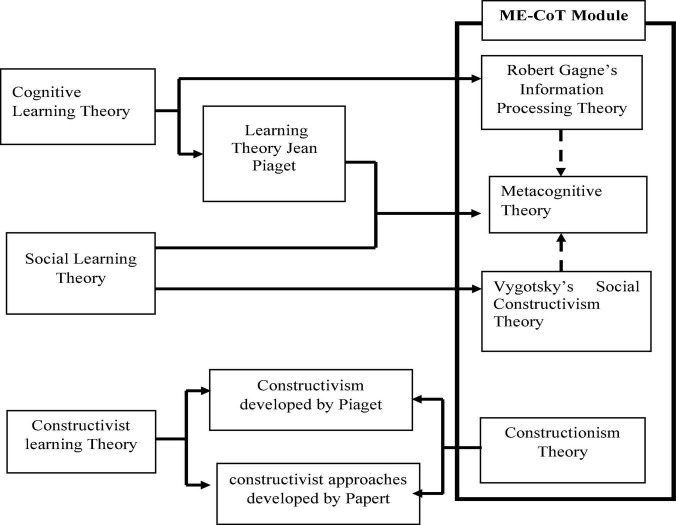
Theoretical integration.

This learning theory can be specified into 4 main theories namely, (i) Theory of Constructivism, (ii) Theory of Cognitive Learning, i.e., Robert Gagne’s Information Processing Theory, and (iii) Vygotsky’s Social Theory of Constructivism, and (iv) Metacognitive Theory. Figure 2 illustrates the learning theory combination that resulted in the creation of the ME-CoT Module Theoretical Framework. ME-CoT is a well-designed module based on the integration of these four main theories.

### Theory of Constructionism

In the analysis and similarities that exist between the constructivist approach developed by Papert and the Constructivism created by Piaget, constructionism is created (Ackermann, 2001). Through programming activities, the ME-CoT module will create output in the form of visual products utilizing ME-CoT Visual Studio. Programming is frequently related to algorithms (Angeli and Jaipal-Jamani, 2018), which involve solving a problem step by step to get the desired result. As a result, programming is a set of activities that can help students develop computational thinking skills through making experiences. Programming is a computing activity that can produce an output with this clear as mentioned by Papert in Theory of Constructionism (Ackermann, 2001). In addition, Constructivist Learning Theory itself also has a very profound impact on this study.

Inquiry-based Learning (Bevins and Price, 2016) is founded on constructivist theory and directly relates to students’ flexibility in choosing and conducting investigations based on their scientific knowledge. Furthermore, the inquiry-based activity approach utilized in teaching and learning is only focused on “learning by doing,” but the theory of constructionism, as described by Papert (1996), prioritizes the development of products as a result of learning, also known as “learning by making.”

Students’ conceptual knowledge can be enhanced via inquiry learning based on the 5E model (Ping et al., 2020). Higher-Order Thinking Skills (HOTS) and Lower Order Thinking Skills (LOTS) are two types of conceptual knowledge. To develop metacognitive awareness, students must master both categories (Burbaite et al., 2018). The constructivist theory provides the foundation for guiding students through the process of mastering metacognitive awareness from a low to a high degree of difficulty. Although, as mentioned in the Theory of Behaviorism, Bloom’s Taxonomy stresses the order of learning sequences according to the amount of difficulty (from low to high). The application of bloom’s taxonomy in this study, however, is based on the guided inquiry learning approach recommended by Constructivist Learning Theory.

### Cognitive Learning Theory (Robert Gagne’s Information Processing Theory)

Cognitive learning theory explains that learning is a change – a change that occurs in the information available in a person’s memory. Furthermore, since this research focuses on metacognitive awareness, which includes sensory memory and long-term memory, Robert Gagne’s Information Processing Theory is essential in the development of students’ metacognitive awareness. Gagne (1970) has sought a variety of superior and perfect ways to ensure learning takes place. A human absorbing and processing information obtained via the senses, according to Gagne (1970), is similar to a computer processing information. Plans are inputting that sensory memory and short-term memory process. The information generated will be stored in long-term memory or used to act with the environment showcasing the learning process. Furthermore, learning occurs when students attempt to comprehend the instructions, and it stimulates the learners’ cognitive abilities through input from the learner’s environment, resulting in change. This procedure is carried out with the assistance of media, which are used as vehicles to deliver important messages (Gagne, 1970). As a result, ME-CoT was purposefully designed around the Cognitive Learning Theory. Based on the ME-CoT activities, students will be assigned a group project to handle the ME-CoT Visual Studio, along with certain instructions that students must comprehend and apply to articulate the Visual Studio and generate the activity product or presentation product. This Module was designed in printed form to serve as a vehicle for delivering adequate knowledge to students for them to complete their assigned work.

### Vygotsky’s Social Constructivism Theory

The application of Vygotsky’s Social Theory of Constructivism occurs due to the presence of interactions in groups or collaborative activities in solving problems. Additionally, metacognitive awareness is linked to Vygotsky’s Social Theory of Constructivism because it provides a platform for students to collaborate with their peers in completing tasks and implementing successful problem-solving techniques in their Proximal Development Zones (SPDs). Students also manage to capture the computational thinking skills through coding activities easily. Students begin to employ cognitive strategies to arrange learning approaches to accomplish tasks that have been provided in the form of problems and inquiries once metacognitive awareness has been developed. By investigating to find answers, students will interact and work with classmates (in the Proximal Development Zone) or teachers. Furthermore, self-reflection displays the use of Vygotsky’s Social Theory of Constructivism in building metacognitive awareness by evaluating the results of tasks.

**FIGURE 3:**
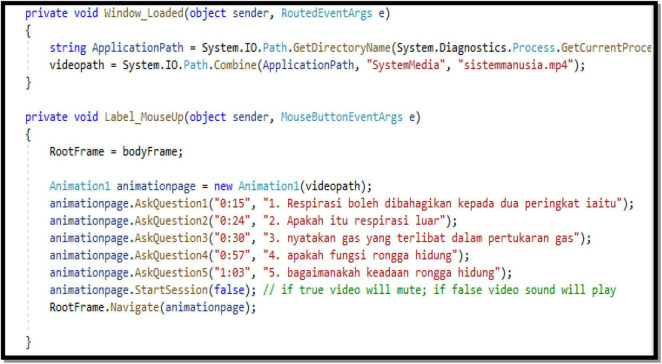
Text-based programming in see, pause, and answer module.

Text-based programming had been categorized as a difficult programming task for students because it is using programming languages such as Java, C+, C++, and C#. ME-CoT Module is developed using a text-based programming tool (Visual Studio with the C# programming language) because kids are already familiar with block-based programming (Scratch) from primary school (Haslina et al., 2018; Kaufmann and Stenseth, 2021). ME-CoT Visual Studio has been crafted using Visual Studio which is user-friendly and well. Surprisingly, ME-CoT Visual Studio was created using very basic programming skills and the C# programming language. Figure 3 shows the task the students did in ME-CoT Visual Studio. The students only need to complete the ME-CoT Visual Studio See Pause and Answer based on the algorithm they constructed in their printed ME-CoT Module during their discussion.

### Metacognitive Theory

After performing a study on a group of preschool and primary school children on their capacity to learn and bind a set of things, Flavell proposed metacognitive theory. In metacognitive awareness, the age and cognitive growth of students are connected. Meanwhile, Flavell’s research was tweaked to look at cognitive aspects in connection to social contact, revealing a link between social engagement and metacognitive awareness (Flavell, 1979; Pressley et al., 1985). Thereby, Metacognitive Theory was found to be strongly connected to Jean Piaget’s Theory of Cognitive Learning (1849–1936) and Vygotsky’s Social Theory of Constructivism (Flavell, 1979).

Metacognitive is referred to as thinking about thinking (Flavell, 1979; Sun, 2013; Mazli Sham and Saemah, 2014; Koc and Kuvac, 2016; Astuti et al., 2017). Thinking is a cognitive talent that incorporates mental activity that develops according to the individual’s level and capacity to adjust at each level with the organization of thinking structure, such as schema, assimilation, accommodation, and adaptation (Piaget, 1972b; Ragbir Kaur Joginder Singh, 2011). The metacognitive theory is rooted in the cognitive dimension (Krathwohl, 2002), cognitive development begins in infancy, and Piaget (1972b) demonstrated that a child’s thinking evolves through stages from infancy to adulthood. Gagne’s information processing theory, on the other hand, helps with teaching-based learning and cognitive processes (Ragbir Kaur Joginder Singh, 2011).

The teaching or design of teaching approaches used by teachers plays an important role in enhancing metacognitive awareness (Çakiroğlu and Er, 2020). Social interaction is very important for effective learning to take place, so this importance shows the relationship between Metacognitive Theory and Social Learning Theory. The formation of Metacognitive Theory has a profound effect on the effectiveness of Metacognitive theory because it acts as an active monitoring and sequential control that occurs consciously over cognitive activity (Flavell, 1979). Thus, Metacognitive is a person’s knowledge and control of thinking and learning activities (Astuti et al., 2017; Chou, 2017). Effective learning will occur when students know what they know and what they need to know to fill the knowledge gap that exists where awareness exists within students. A person’s awareness of what is known and what is not known is also known as metacognitive. Metacognitive strategies are methods used by students to increase awareness of the process of thinking and learning that takes place in the students themselves. Metacognitive awareness can help students control their minds by planning, monitoring, and evaluating the information they have learned (Koc and Kuvac, 2016; Kyairaniah et al., 2017; Harrison and Vallin, 2018).

Students are responsible for their knowledge. Metacognitive awareness must be seen in terms of Planning, Monitoring, Evaluation, Information Management Strategies, Debugging Strategies, Declarative Knowledge, Procedural Knowledge, and Conditional Knowledge. Therefore, in this study metacognitive constructs consisting of aspects of Planning, Monitoring, Evaluation, Information Management Strategy, Debugging, Declarative Knowledge, Procedural Knowledge, and Conditional Knowledge (Schraw and Dennison, 1994; Harrison and Vallin, 2018) will be assessed using a questionnaire instrument the Metacognitive Awareness survey constructed by (Schraw and Dennison, 1994).

**FIGURE 4:**
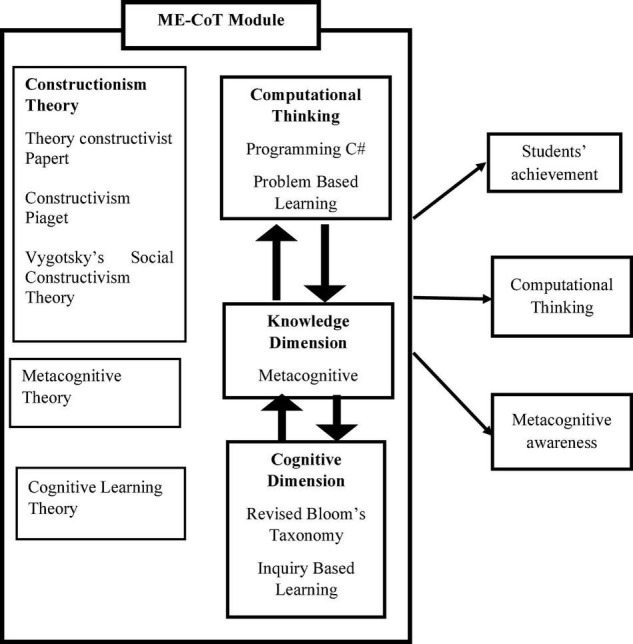
Conceptual framework of the ME-CoT.

The study’s conceptual framework is given in Figure 4, which is made up of a combination of Learning Theories. Table 1 shows, the Framework of Computational Thinking as a Problem-Solving Process (Kalelioglu et al., 2016) and the revised Bloom’s Taxonomy and Computational Thinking (Revised Bloom’s Taxonomy and Computational Thinking Model) are used to explain how to apply computational thinking and foster metacognitive awareness. The use of Learning Theory, as well as the combination of the Computational Thinking Framework as a Problem-Solving Process (Kalelioglu et al., 2016) and the Revised Bloom’s Taxonomy and Computational Thinking Model, to ensure effective learning in the classroom in terms of improving students’ achievement, computational thinking, and metacognitive awareness among Form 4 Biology students.

### Metacognitive Empowerment by Computational Thinking Visual Studio

Metacognitive Empowerment by Computational Thinking (ME-CoT) module is crafted using Morrison, Ross, Kalman, and Kemp (MRK) model. MRK model is a spiral model which is a very suitable and flexible model to be used in the classroom. ME-CoT is shaped based on the instructional design model which emphasizes nine main elements such as instructional problems, learner characteristics, task analysis, instructional objectives, content sequencing, instructional strategies, designing a message, development of instruction, and evaluation instrument. However, the content of the ME-CoT Module is aligned with the need of the educational Theory. ME-CoT Visual Studio is a text-based programming tool (Visual Studio with C# programming language) that eliminates the block-based programming (scratch) that the pupils were taught in primary school. As a result, the researcher was able to construct the ME-CoT Module, which critically reflects on content knowledge and has a positive impact during classroom implementation. Furthermore, ME-CoT Visual Studio was created with Visual Studio, which is user-friendly and has a design that allows for the inclusion of images and videos that are essential to Biology education.

#### See Pause and Answer Module

This “See, Pause, and Answer” module is a type of group project that instructs students on how to use video to generate questions and create a quiz-based activity product using the See, Pause, and Answer module. This module will be sent to other members of the group to assist them in answering the questions and to assist teachers in identifying students’ misconceptions of the topic of study. This module was built using a combination of standard biology content knowledge and programming skills. Students will study and comprehend the content of the Respiratory System in Humans and Animals by viewing videos, and they will get better knowledge by preparing questions based on the video they have viewed.

**FIGURE 5:**
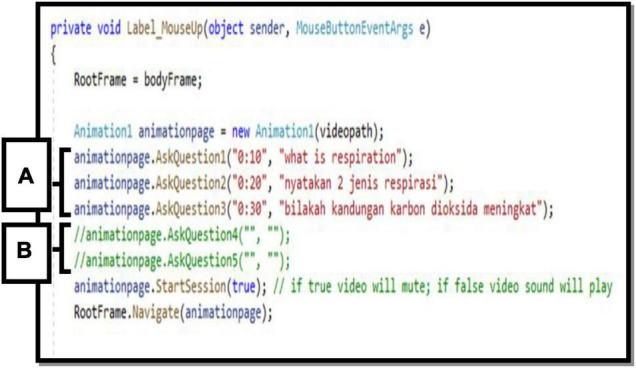
C# Programming sheet in visual studio.

Figure 5 shows the task that the students completed in ME-CoT Visual Studio. The students only need to complete the ME-CoT Visual Studio See Pause and Answer depending on the algorithm they generated in their printed ME-CoT Module. The algorithm consists of the time when the video should stop to pop out the question. In the brackets provided, students should write the time and questions. Only the words in red should be altered by the student.

The question instructions are presented during the product launch in the section labeled A, when the question comment is active. The portion labeled B, on the other hand, is a line of inactive questions. Students must use the symbol (//) to trigger a question. Students must also pay attention to the directions offered to them.

*e.g., Animation.startsession (true); // if true video will mute; if false video sound will play*

“True” indicates that the video included in the ME-CoT Visual Studio will play without sound or music, while “false” indicates that the video will work with audio.

Using Cognitive Learning Theory, students will begin to grasp computational thinking abilities through the use of the C# programming language throughout product development tasks. It has been demonstrated that students may make a product by creating the activity product, as indicated by the Theory of Constructionism. The product is assessed through debugging activities before being distributed to students in other groups to answer and learn more about the Respiratory System in Humans and Animals. This also allows students to detect faults in their understanding of the topic. This content employs metacognitive theory. Students will gain knowledge through making errors. Students can develop metacognitive awareness by identifying and improving deficiencies through the exercises given. The SPA Module “See, Pause, and Answer” is remarkable in that students must observe and comprehend the video that has been presented. The students should next create an activity product containing the questions to distribute to other groups of students. The creation of questions based on information from the video is an important tool for students to develop metacognitive awareness.

#### Drag and Drop Module

The Drag and Drop Module is a type of group assignment that instructs students on how to identify each respiratory component and its functions in the chapter on the Human and Animal Respiratory System. Students are expected to create activity items to assist other group members in answering the questions and to assist teachers in identifying students’ misconceptions about the topic of study. This module was built using a combination of standard biology content knowledge and programming skills. The Respiratory System in Humans and Animals will be taught and understood by students.

Based on the topic and facts taught, students will create an activity product. Students begin to use Cognitive Learning Theory when they get a better understanding of content standards. Students will next use images or graphics to create a “Drag and Drop” activity product.

In the ME-CoT Visual Studio Drag and Drop, the value of images on the topic of Biology is highlighted. Students will begin to grasp computational thinking abilities through the use of programming in the C# language during the activity product development.

Students learn how to recognize the ME-CoT Visual Studio Drag and Drop in greater detail, how to integrate photos and create responses in the Drag and Drop Software Module, and how to write a clue text in this part. The “Drag and Drop” activity demonstrates that students may construct a product, as indicated by the Theory of Constructionism. The product is assessed through debugging activities before being delivered to other groups to answer questions and learn about the Respiratory System in Humans and Animals. Additionally, utilizing ME-CoT Visual Studio Drag and Drop to create activity products allows students to pinpoint faults in conceptual knowledge. In this case, the metacognitive theory is used. Students can develop metacognitive awareness, or the ability to identify and fix deficiencies, by participating in tasks given by the ME-CoT Visual Studio Drag and Drop.

#### Speak Out Module

This “Speak Out” Software Module is a type of Visual Studio software module for expressing students’ knowledge of the area being studied. This module teaches pupils how to spontaneously talk or articulate material they’ve studied. The “Speak Out” Software Module is unique because it can transform the audio produced along with the picture into video. Teachers evaluate the conclusions or summaries of the learning areas that have been covered using the module as a presenting output. This module was built using a combination of standard biology content knowledge and programming skills. Through classroom activities, students will study and grasp the topic of Respiratory systems in Humans and Animals.

The field of Biology is enriched with images that require a deeper understanding. Students should be able to utilize images to express concepts, processes, and information clearly and concisely. The concept of Cognitive Learning Theory plays a significant part in knowledge construction. Students will begin to acquire computational thinking abilities throughout the creation of the “Speak Out” output by using the C# programming language. Students can create a presenting product after completing the “Speak Out” Software Module, as described in Theory of Constructionism. The presentation product created by the “Speak Out” Software Module is a valuable tool for assessing students’ knowledge of the Respiratory System in Humans and Animals topic. Students’ inability to master the topic is indicated by errors in image interpretation. When a teacher or a coworker provides feedback, the pupils may be able to fix their errors. From the programming of the product through the assessment of the product derived from the Speak Out Module, Metacognitive Theory is used in this context. As a result, students begin to develop metacognitive awareness by planning, monitoring, and analyzing the provided activity. Furthermore, when students complete the assignment, they begin to enrich themselves with the subject knowledge they have acquired. Students will discover and correct problems at each level of the programming activity so that the presentation product launched can produce the desired results.

The module was developed by integrating Problem Solving Computational Skills, and these skills will arouse students’ metacognitive awareness. The below table gives a clear explanation of the impact of using the ME-CoT Module on students. Each item found in the construct was able to assess students’ metacognitive awareness through the application of the ME-CoT Module. Table 2 shows the application of the ME-CoT module to foster metacognitive awareness in students. Students who use the Me-CoT Module have the potential to make plans by speeding up the learning rate as well as setting specific goals before starting programming activities as set out in the ME-CoT Module. In addition to careful planning, students monitor themselves every time they make a programming activity. Students will consider several options for completing activity product assignments and presentation products. To further strengthen metacognitive awareness students should be able to make assessments. Self-evaluation and the success of an activity product or presentation product that has been built through programming activities. Students need time to understand something new information, especially information related to information management or information. Students will try to slow down the activities so that they can focus on important information. While using the ME-CoT Module, students can draw their examples and draw diagrams or pictures to ensure that they understand the new information they have learned.

**TABLE 2:** Fostering metacognitive awareness by implementing the ME-CoT Module.

Metacognitive awareness	Fostering metacognitive awareness in the application of the ME-CoT Module
Planning	Students can plan in terms of time while preparing activity products. In addition, students should focus on every available information while building the activity product. Students should ascertain the type of product they are producing by providing an algorithm. Students read each step and understand each step that exists before starting the product production activity. Students should prepare the activity or presentation product within the time allocated for them.
Monitoring	Students should examine each step and ensure that each step is followed to achieve the goal. Revisions are very important to ensure that activity products and presentation products are successfully launched. Students should examine and focus on each of the options available in the formation of activity products and presentation products. For example, in the Speak Out module, students should provide algorithms in the image-based print module and provide image-related information. Next, the students should prepare a video presentation product. Students should use correct and accurate strategies to ensure that the video produced is accurate based on the images as well as the questions that have been posted. Students can directly test their level of achievement when students are exposed to a new technique in Visual Studio.
Evaluation	The evaluation is highly prioritized in the application of the ME-CoT Module. Students can assess their level of achievement after preparing an activity product. To further strengthen the understanding of students will use the results of the activity to assess their understanding of the students. Students will make sure they understand the content contained in the video that has been given before preparing the activity product. The See Pause and Answer module is a module that helps students to understand the whole information or content so that they can construct questions and answers based on the video before producing the activity product.
Information management strategy	Students must understand each step to produce a quality activity product or presentation product. In addition, to focus on image accuracy, students should focus on the image’s size. Students will focus on each piece of information and each step to successfully launch an activity product or presentation product. Besides that, during the use of the module students have the potential to produce their algorithms based on the steps that have been given as well as students will produce their examples and their way of working to produce a presentation product.
Debugging	Students also get help and guidance from teachers or colleagues in understanding the activities that have been given. In addition, to examine the effectiveness of measures, students also have the potential to change the technique of each activity according to their suitability, for example when producing a product using programming learners will face various challenges, students who easily understand the concept will continue to produce products, while those who do not understand may need guidance and should make sketches or stop and review any new information that is less clear.
Declarative knowledge	While producing activity products, students can train themselves to know the important information that students should. Because, lesson content is the information available in textbooks and reference books students will read and understand as available, but students will focus more on computer science components such as programming. Students will try to understand the programming component’s intellectual strengths and weaknesses. Students will also ascertain the objectives and information required by the teacher while producing the activity product or presentation product. Students are also able to control and assess their level of understanding of new information introduced to them. Students are also able to increase their interest in the topics studied when a new context is introduced to them.
Procedural knowledge	Students will provide two activity products and one product or presentation. Students will ensure each product is produced strategically and correctly. For example, students will follow each step in producing a product in their way. However, each strategy that the students use is based on their understanding and suitability to produce activity products and presentation products.
Conditional knowledge	Students need early exposure to the topics they are studying. Thus, the ME-CoT Module does not depart from the context emphasized in Vygotsky’s Theory of Social Constructivism, where students have exposure to Biology content topics since primary school and during lower secondary, while for computer science, students are used to the size of the image and resizing images since lower secondary. Students are also good at using computers with basic information. However, programming using the C# programming language is new information introduced in a very simple and easy way through the ME-CoT Module. To successfully launch activity products and presentation products, students will always be highly motivated by using students intellectual strengths as well as balancing weaknesses. Students also know the appropriate and most effective strategies for producing activity products or presentation products by using the ME-CoT Module.

## Evaluation and Results

Metacognitive Empowerment by Computational Thinking (ME-CoT) is a well-built tool that has been validated by seven professionals in the area. Following the validation, a preliminary investigation was carried out to determine its dependability. In this study, the general assessment was made using the Fuzzy Delphi measure where to obtain the expert agreement in determining the suitability of the ME-CoT module applied in the Biology form 4 classroom.

### Validity

The content validity of the Visual Studio software module ME-CoT has been evaluated by seven experts. The Visual Studio software module ME-CoT is divided into 3 Modules. Each Module is carefully evaluated by experts. According to Mohd Sidek and Jamaludin (2005), if the content validity coefficient is equal to 70% or more, then the module built or produced has high validity. All items recorded a coefficient value exceeding 70%. According to Jamaludin (2008) and Kusuma et al. (2017), the value of the reliability coefficient of a measuring instrument or the activity of a module is the minimum that can be adopted is 0.50. Jamaludin (2012) has stated that the reliability coefficient value must be at least 0.60. While Jamaludin (2012) has stated that the value of the reliability coefficient of the measuring instrument or module activity is 0.90. Therefore, if the value of the reliability index of this module is between two minimum reliability values of 0.50 and a maximum of 0.90, then this module is acceptable and applicable. Meanwhile, the overall coefficient of the ME-CoT module also recorded a high value of 0.90. Although the content validity and coefficient have a good value, Biology instructors and students have provided several recommendations to help develop and improve the ME-CoT Module.

The Fuzzy Delphi sampling used is purposive sampling and criterion sampling, this is because each expert or sample is selected based on the purpose, based on the experience of experts in the field studied. Meanwhile, this method is categorized as judgment sampling because individual judgment is used to select the study sample based on the researcher’s knowledge and study needs. There are seven main steps in implementing the Fuzzy Delphi method. The first step is the determination of expertise. Expert expertise is based on, qualifications, individual character, ability to compare differences, consistency and reliability, time, and experience. Meanwhile, tenure and teaching background recognition from certain experts and bodies, awards and certifications from certain institutions Educated student achievement and performance benchmarks are also classified as benchmarks for the expertise of an expert in a particular field of study (Ramlan and Ghazali, 2018). In the study, the selected experts are individuals who are skilled in the field of programming, STEM field, Biology Education, and HOTS. The next step is the second step, namely, determining linguistic variables based on the Triangular Fuzzy Number (determining linguistic scale). The second step involves converting all linguistic variables into fuzzy triangle numbering. Likert scale data obtained in the first stage were analyzed using the Excel program for more neat scheduling. All Likert scale data is converted into a triangular Fuzzy number. The triangular Fuzzy Number represents the values of m1, m2, and m3 (Ramlan and Ghazali, 2018). The number will be written in the form (m1, m2, m3). The value of m1 means the minimum value, the value of m2 indicates a reasonable value and the value of m3 represents the maximum value. The three values of m1, m2, and m3 are used to produce a fuzzy scale. In this study, the 5 -point Fuzzy scale agreement level was used. Study data were then scheduled to obtain Fuzzy values (n1, n2, n3) as well as average Fuzzy values (m1, m2, m3) to obtain threshold values, expert percentage, defuzzification, and item ranking.

The third step is the distance determination step to identify the value of Threshold “d.” The threshold value of “d” which is less than or equal to 0.2 indicates that the evaluated module is categorized as the expert agreement has been reached (Ramlan and Ghazali, 2018). The reading of the mean value of d in this study shows 0.153 where it is at a value below 0.2 then all the experts reach an agreement on the item referring to the general evaluation of the ME-CoT Module. Next, is the determination of the percentage of group consensus which is the fourth step, where the overall consensus (group consensus) should exceed 75% (>75%) for each item. If each item is equal to or exceeds 75% then each item has reached expert consensus. All items have passed 75% which indicates all items were accepted. General evaluation of the Me-CoT Module recorded the overall percentage of expert agreement is at 92% agreement value which is more than (>75%) means that the general evaluation items of the mE-CoT module have been accepted the conditions of expert agreement on general evaluation items of ME-CoT Module.

The next step is the fifth step which identifies the α-Cut value and the sixth step by identifying the α-Cut defuzzification value (average of fuzzy response). The α-Cut defuzzification value (average of fuzzy response) must exceed 0.5 (>0.5) and if the α-Cut value is less than 0.5 then the item should be dropped and the item does not qualify. The ranking of each item is sorted from the highest fuzzy rating value to the lowest value. Items 13, 11, and 9 show the highest α-Cut value of 0.767 with the first position. This shows that experts agree that, ME-CoT Module is a module containing activities that cultivate the cognitive domain and knowledge domain of students, built based on the new curriculum revision 2017 as well as suitable for use by students aged 16 to 17 years. Meanwhile, items 2,4,5,10, and 12 recorded an α-Cut value of 0.733 with the second position. This shows that the ME-CoT Module can cultivate computational thinking, help students master the field of learning, guide students to solve problems systematically, cultivate inquiry-based learning and the ME-CoT Module also focuses on “learning by making”. Items 1, 3, and 7 also recorded an α-Cut value of 0.700 which is the item on the position.

This research included fourteen biology students from one school in one district in Malaysia. It will be examined how programming with Visual Studio helps students in gaining content knowledge of Biology Education. Each module has been assessed separately to have a better understanding of its usability and reliability.

The quasi-experimental study has a sample size of fewer than 30, with 15 students in the experimental group and 14 form 4 Biology students in the control group. According to the findings of the research, the pilot study sample should account for 10% of the total sample size for the real study (Lynne, 2008). With the number of pilot study samples containing 10 samples, the purpose of a pilot study that did not include the construction of new instruments but examined the performance of instrument items created by other researchers with new populations was appropriate (Isaac and Michael, 1995). However, in this study, the pilot study sample included 14 students, with more than 10% of the sample participating in the pilot study. A total of 14 Biology students participated in this pilot research. Furthermore, due to the COVID-19 pandemic, only 14 students were allowed to be used for the pilot study [permission from EraS (Ministry of Education, Malaysia) and the Secondary School Principal]. This study was conducted after receiving the permission letter from *Educational Research Application System* ERaS, the ethical approval was granted by the Ministry of Education Malaysia [Reference number: KPM.600-3/2/3-Eras (11306)].

Besides that, strict SOP in conducting the educational research in school restricted the number of students involved in the pilot study. This study is conducted 3 weeks after the reopening school announcement which had been made by the Ministry of Education, Malaysia. Due to that issue, a suitable statistical analysis had been carried out to analyze the pilot study finding in the reliability of the module.

### Preliminary Study for Reliability

Reliability refers to the consistency of a measure while the Cronbach Alpha value is an estimate of the internal consistency of each item in a study instrument (Isaac and Michael, 1995; Hertzog, 2008; Lynne, 2008). The alpha coefficient depends on the variance of the items and the correlation between the items. To ensure the reliability of the measurement tool or study instrument is consistent, the alpha sedative value should exceed 0.75 for a pilot sample of 25 to 40 people. Since the study sample was less than 30 students then, the consistency of the measurements, Pearson’s r was calculated to provide an estimate of reliability (Odom and Morrow, 2006) to further strengthen the findings of the study. There are three, reliability coefficients involving Pearson’s r namely (i) stability coefficient, (ii) equivalence coefficient and, (iii) objectivity coefficient (Odom and Morrow, 2006). In this study, a stability coefficient is used where the consistency index is between two test times. The first evaluation was conducted immediately after the implementation of the pilot study while after 2 weeks the second evaluation was conducted. Estimates of the consistency index between the two tests were identified using Pearson’s correlation coefficient r. Normally a value of Pearson’s correlation coefficient r of at least 0.70 is acceptable stability but according to Hertzog (2008), the value of Pearson’s correlation coefficient r must be above 0.80 is highly recommended for samples with small pilot studies. The correlation Strength Scale by Cohen et al. (2002) was used in the study. Table 3 shows the Correlation Strength Scale. The less quality measurement was used to identify the reliability due to the number of samples used for the pilot study is less than 15, which is only 14 students were used due to the restriction.

**TABLE 3:** Relationship strength according to the value of the correlation coefficient.

Size of correlation coefficient (r)	Correlation strength
± 0.81 to 1.00	Very strong
± 0.51 to 0.80	Strong
± 0.31 to 0.50	simple
± 0.21 to 0.30	weak
± 0.01 to 0.20	Very weak

#### Reliability of Metacognitive Empowerment by Computational Thinking Visual Studio

The ME-CoT module is divided into two main parts, which are ME-CoT Visual Studio and the ME-CoT print module. Stability coefficients are used to determine the reliability of the Visual Studio ME-CoT Module. The first evaluation was conducted right after the pilot project was launched, and the second evaluation took place two weeks later. Estimates of the consistency index between the two tests were identified using Pearson’s correlation coefficient r. Normally a value of Pearson’s correlation coefficient r of at least 0.70 is acceptable stability but according to Hertzog (2008), the value of Pearson’s correlation coefficient r must be above 0.80 is highly recommended for samples with small pilot studies.

Table 4 shows that there is a significant relationship between the first evaluation and the second evaluation of the See, Pause, and Answer Module with a value of *r* = 1.000 Sig = 0.000 (*p* < 0.005). The strong correlation coefficient index indicates the high usability and reliability of the ME-CoT Visual Studio module. The See, Pause, and Answer module, is the ME-CoT Visual Studio module that involves activities to produce activity products. The activity products produced by students involve various skills recommended in computational thinking which are highly emphasized in the ME-CoT module. Students must understand the content presented in the instructional video, then students must provide an algorithm that contains 3 questions (for a pilot study) along with the time for the questions displayed in the video. In addition, evaluation is emphasized in this study, where students prepare questions with answers. That answer is to be filled into answer.txt. in the ME-CoT Visual Studio module. This evaluation has a very important impact not only on computational thinking but on creating metacognitive awareness as well. Once the student prepares the activity product the student should launch the product, at this stage the student will identify their mistakes in building the product. Usually, this error occurs when students prepare the images (image size) and insert images into the ME-CoT Visual Studio. However, planning, monitoring, evaluating as well as strategically managing information applied by students can directly address the problem of a product launch by the students.

**TABLE 4:** Pearson’s correlation coefficient index r ME-CoT Visual Studio.

Relation	Second evaluation						Interpretation
	See Pause and Answer		Drag and Drop Module		Speak Out Module		
	r	Sig	r	Sig	r	Sig	
First evaluation See Pause and Answer Module	1.000	0.000					Very strong
First evaluation Drag and Drop Module			0.967	0.000			Very strong
First evaluation Speak Out Module					0.974	0.000	Very strong

Secondly, it is the Drag and Drop Module, the relationship of the first evaluation with the second evaluation of the Drag and Drop Module with a value of *r* = 0.967 Sig = 0.000 (*p* < 0.005). The strong correlation coefficient index indicates the high usability and reliability of the ME-CoT Visual Studio module, especially the Drag and Drop module. This Drag and Drop module, focus on the images especially the characteristic of respiratory structure in human and animals. Pupils needed to draw the respiratory structure of the given task, then they need to search for the related picture on the internet. This activity took time. so, the students are advised to spend time wisely while searching and editing the picture. Then, the students were asked to crop the picture and name the images. Pupils then, list out the images and its characteristic which is known as an algorithm. After collecting the pictures needed, the students will produce the product activity using the Drag and Drop Module. Students will fill up the name of the image in Mainwindow.xaml (ME-CoT Visual Studio module) then the students will fill up the answer text and clue text. It shows that students need to follow the instruction given in the module and create the product activity. Students took time in collecting and downloading the images. Although students find difficulties in downloading the pictures, students were well equipped with the knowledge of editing and resizing images.

Lastly, Speak Out Module shows the relationship of the first evaluation with the second evaluation of the Speak Out Module with a value of *r* = 0.974 Sig = 0.000 (*p* < 0.005). The recorded findings have a very strong relationship based on the Relationship Strength Scale of Cohen et al. (2002). The strong correlation of Pearson’s r the stability of the pilot study was above 0.95 which recorded a very encouraging value of above 0.80 for the small group of pilot studies (Hertzog, 2008). This finding revealed that students managed to create the presentation product. Students were given a question, then they need to read and understand the question. Then they must find the related images then talk about the question and relate it with the picture. Before beginning the speaking task, students need to list out the points and the number of images as an algorithm. Pupils will speak out the information and launch the product. The product will be presented as a video. Each group had created a video. Students were excited about using this Speak out module because they can hear their voices when the video was played in front of the classroom.

Meanwhile, to see the effectiveness of the ME-CoT module in improving student achievement in topics, fostering computational thinking and metacognitive awareness, a screening test was conducted during the pilot study. Assessments were made before and after the use of the ME-CoT Module.

The Statistical results of the Wilcoxon Signed Ranks Test in Table 5 showed that there were differences in achievement test scores in the Respiratory System in Humans and Animals, before and after the use of the ME-CoT Module (*p* = 0.001, *p* < 0.05). The results of the analysis clearly showed that the mean value of the positive rank (mean rank = 7.50) was higher compared to the mean rank for the negative rank (mean rank = 0). Descriptive statistical data mean achievement test score (pre) for the pilot group was recorded as mean = 25.86 which is less than the mean of the post-test of the pilot group which is mean = 46.00. To determine the reliability of the computational thinking questionnaire instrument as a problem solution, Pearson’s r correlation coefficient index was used with 2 assessments. The first assessment was on the day of the pilot study while the second assessment was conducted two weeks after answering the first assessment, Table 6 shows the Pearson’s r coefficient index of Computational Thinking as Problem Solving.

**TABLE 5:** Wilcoxon Signed Rank exams for pre and post exams for achievement scores.

Student’s achievement	*N*	*M*	*SD*	Median	Z	Sig
Pre	14	25.86	6.225	24.5	−3.297	0.001
Post	14	46.00	10.735	50		

**TABLE 6:** Pearson’s correlation coefficient index r Computational Thinking as a problem solving.

Relation	Second evaluation computational thinking as a problem solving		Interpretation
	r	Sig	
First Evaluation Computational Thinking as a problem solving	0.995	0.000	Very strong

There was a significant relationship between computational thinking as a solution to the first assessment problem with the second assessment of students with a value of *r* = 0.995 sig = 0.000 (*p* < 0.005). Its strength is 0.995 which indicates a very strong relationship. Relationship strength is based on the Relationship Strength Scale Cohen et al. (2002). While Table 7 shows a significant relationship between metacognitive awareness of the first assessment with the second assessment of students with a value of *r* = 0.900 sig = 0.000 (*p* < 0.005). Its strength is 0.900 which indicates a very strong relationship.

**TABLE 7:** Pearson’s correlation coefficient index r metacognitive awareness.

Relation	Second evaluation metacognitive awareness		Interpretation
	r	Sig	
First Evaluation Metacognitive Awareness	0.900	0.000	Very strong

The findings of the pilot study showed that there was a noticeable effect of change before and after using the ME-CoT module. Despite very slight changes in achievement in test scores, the use of the ME-CoT Module can be seen to be effective. Meanwhile, a very strong relationship can be seen among students in terms of fostering computational thinking skills and metacognitive awareness.

In this study, the researcher used assessment test questions to analyze student achievement, while computational thinking as a problem-solving instrument (Yağcı, 2019) and Metacognitive Awareness Inventory (Schraw and Dennison, 1994; Harrison and Vallin, 2018) were used to analyze students’ computational thinking and metacognitive awareness. The study’s findings revealed that students who utilized the ME-CoT module performed better academically. Meanwhile, a substantial correlation demonstrates that computational thinking skills and metacognitive awareness are both very reliable. In the actual study, the development of computational thinking abilities and metacognitive awareness will be examined in depth.

#### Printed Metacognitive Empowerment by Computational Thinking Module

In addition to the Visual Studio Software Module, the printed material ME-CoT Module was also evaluated for its overall reliability index. Table 8 shows the Pearson’s Correlation Coefficient Index for the ME-CoT Module. The findings of the pilot study showed a significant relationship between the Printed ME-CoT module of the first assessment with the second assessment of students with a value of *r* = 0.772 Sig = 0.001 (*p* < 0.005). A value of Pearson’s correlation coefficient r is less than 0.80 is not recommended. Nevertheless, a reading of 0.772 is reading above 0.70 acceptable stability (Cohen et al., 2002; Hertzog, 2008).

**TABLE 8:** Pearson’s correlation coefficient index ME-CoT Module.

Relation	Second Evaluation Printed ME-CoT		Interpretation
	r	Sig	
First Evaluation Printed ME-CoT	0.772	0.001	Strong

Meanwhile, students who used the ME-CoT module also displayed feelings of happiness and fun when using the ME-CoT Module in Lessons. Students also stated that they enjoyed listening to their voices as well as their explanations on the topics given which automatically increased students’ self-confidence in learning Biology. Meanwhile, not only does it help self-learning, but the ME-CoT Module also helps peers to prepare activity products to assess peers’ mastery of the units studied also indirectly attracts the attention of students. The findings of the pilot study showed that there was a noticeable effect of change before and after using the ME-CoT module. despite very slight changes in achievement in test scores, the use of the ME-CoT Module can be seen to be effective. Meanwhile, a very strong relationship can be seen among students in terms of fostering computational thinking skills and metacognitive awareness.

### Views and Suggestions of Biology Teachers

The ME-CoT Module was tested at a school under the auspices of PPD Jempol and Jelebu on a Form 4 Biology teacher and 14 students. The Me-CoT module was implemented in the classroom by the Biology teacher. The ME-CoT Module is implemented by Biology teachers under the supervision of researchers. The opinions and ideas of teachers are solicited and studied to develop the ME-CoT module. Table 9 illustrates the opinions and ideas of Biology teachers who helped with the ME-CoT Module usability test.

**TABLE 9:** Teacher’s views and suggestions.

Criteria	Teacher’s views and suggestions
Advantages	∙ The Me-COT module is appealing and simple to operate. Contains colorful images that draw pupils’ attention. ∙ Students can master the material of the course because they construct their questions, answers, and explanations. ∙ Group activities are a lot of fun for students. Students may create their activity items and are delighted to see their percentage marks.
Weakness	∙ Students take a long time to grasp the concept of programming in the early stages of introduction; nevertheless, a quick video presentation on each step of programming helps students master the processes of programming.
Suggestions for improvement	∙ Please include a topic header on each page for easy reference. ∙ Students can be provided a video presentation of the visual studio programming module’s essential material, which can also be attached in softcopy form. ∙ In the module, provide a student information page. Students can save time by using the module if appropriate photos and sizes are provided in one folder.

Adding a heading to each page sheet is one of the enhancement ideas that help students when using the ME-CoT module. Opening and searching pages from a list of contents take a lengthy time for students. Meanwhile, students would be able to spot problems more readily if module usage videos are distributed to them in softcopy form. Aside from that, the teacher had pointed out a page for students to record their names in the module, which should be added to the students’ module. In addition, an image folder and a video folder were proposed to be added to the module to assist students in making the most use of the ME-CoT Module. This module, according to the Biology teacher, is very interesting not only because it encourages students to master the content of Biology, but also because it helps students pay attention to both the subject of Biology and the field of programming, which has the potential to produce high-quality and interesting learning products.

The ME-CoT module is developed based on the principles interlinked between the three main Learning Theories, namely, Cognitive Learning Theory, Social Learning Theory, and Constructivist learning Theory. If traced, the ME-CoT module was developed by combining four learning theories namely; Robert Gagne’s Information Processor Theory, Metacognitive Theory, Vygotsky’s Social Theory of Constructivism, and Theory of Constructivism. As proposed in the Theory of constructionism, the result of the ME-CoT module is three products consisting of two activity products and one presentation product. Meanwhile, learning by making mentioned by Papert in Theory of constructionism can be seen clearly in this study. This study produces an output (activity product or presentation process) in the form of an assignment in technology media through programming activity (C#programming language). This can show the effectiveness of the Me-CoT module in integrating computational thinking into learning. Students ‘thinking can be constructed with systematically arranged problem-solving activities. In addition, students’ thinking can be guided through the production of new products using technological tools (Kafai and Burke, 2014). Besides that, this study emphasizes the need for coding is not for making unnecessary products, but the product created should have value such as creating games, stories, and animations that can be shared with others (Kafai and Burke, 2014). With this, the Theory of constructionism which focuses on the formation of products through programming activities that develop computational thinking is accurately classified as training students to think, as well as fostering metacognitive awareness in students.

Furthermore, the metacognitive theory is closely related to thinking about thinking (Flavell, 1979; Sun, 2013; Mazli Sham and Saemah, 2014; Astuti et al., 2017). Thinking involves a cognitive activity which is a change in mental activity that develops according to the level and ability of students. Metacognitive awareness guides students to think from a low level to a high level. This thinking also guides students in improving achievement (Mazli Sham and Saemah, 2014; Mohamad Masrizan, 2019). In this study, students plan each activity by setting a time and finding information. Next, students begin to monitor the activities they have done or review their assignments. After monitoring, students will make an assessment. This process is continued with the application of information management that has been obtained. According to Metacognitive Theory, students begin to think of identifying weaknesses and finding solutions then students have begun to seek help from teachers or peers. As students are exposed to new information students begin to analyze their intellectual strengths and weaknesses, especially in this study the application of programming can guide students to focus on the programming component. Meanwhile, appropriate strategies are highly focused by students on ensuring that learning takes place in students. At the same time, students can master student content more efficiently as well leads to improvement in achievement. Thus the ME-CoT Module does not deviate from the context emphasized in Vygotsky’s Theory of Social Constructivism, where students have exposure to Biology content topics since primary school and during lower secondary, while for computer science, students are sensitive about image size and resizing image since lower secondary. Students are also good at using computers with basic information. However, programming using the C# programming language is new information introduced in a very simple and easy way through the ME-CoT Module. Vygotsky’s Social Theory of Constructivism Learning experiences can be built-in in students.

Meanwhile, the third theory namely Vygotsky’s Theory of Social Constructivism is one of the most innovative learning theories in the 21st century (Kozulin, 2015) at the point of emphasis in the production of the ME-CoT Module. The interaction of historical, social, and personal experiences forming a psychological consciousness is an important foundation in Vygotsky’s Social Theory of Constructivism (Vygotsky, 1979b). Social interaction is very important in the ME-CoT module. Where students interact with each other in groups and carry out group activities to produce activity products or presentation products. In addition, guidance from teachers or peers is also preferred, whereas students who have difficulty in solving a problem need guidance from teachers or peers (Pressley et al., 1985). Furthermore, the selection of the programming language also played a role in this study. To ensure that students do not experience cognitive load, the ME-CoT module using a programming language is in line with Vygotsky’s Social Theory of Constructivism where new concepts are developed in the Proximal Development Zone. Based on the study by Stripeikaitė (2017) showed that students who use C-Syntax master programming in more depth. In addition, students who are exposed to C-Syntax programming (such as C ++, Java, and C#) can master the programming language more easily compared to students who learn block-based programming such as Scratch (Stripeikaitė, 2017). Furthermore, according to Krpan et al. (2017), the use of C# and Python languages has been widely introduced among primary and secondary school students. Both programming languages are very popular and have the advantage of being transferred or expanded to other languages (Krpan et al., 2017). Therefore, the C# programming language used in this study is flexible and has been introduced since primary school abroad (Lin and Weintrop, 2021), so it is undeniable that the C# programming language is very appropriate and is in the Proximal Development Zone proposed in Vygotsky’s Social Theory of Constructivism.

In addition, Robert Gagne’s Information Processor Theory is one of the theories that formed the basis of the development of this ME-CoT module because the principles and laws of the theory that emphasize the cognitive development of a student. Learning is closely related to the changes that occur in the information available in the memory of students. Students ‘memory develops in line with the development of students’ metacognitive awareness because this learning theory involves sensory memory and long-term memory. Meanwhile, technological tools such as laptops and Visual Studio software modules used in enhancing long-term memory are closely related to activity product production activities and presentation products produced by students through each level of problem-solving suggested in computational thinking skills. Meanwhile, each skill trains students to think of ways to solve problems systematically while fostering metacognitive awareness in the students themselves. When students try to understand a problem to find a solution then students use long-term memory that exhibits the occurrence of the learning process. The use of technological tools (C#programming) guides computational thinking and ensures that the learning process occurs through metacognitive awareness and in turn plays a role in improving student achievement.

## Limitations, Future Direction, and Recommendation

This study, like any other, has limitations. To begin with, this research is only focused on problem-solving computational thinking skills. As a result, the ME-CoT Module was built around six main problem-solving computational thinking skills. Those problem-solving computational skills have a close correlation to the construct of metacognitive awareness, and they help students develop metacognitive awareness. The ME-CoT is based on the respiratory topic, which has been identified as one of the most crucial topics for students to master to perform well on the general Biology Exam. Abstraction, decomposition, algorithmics, pattern recognition, modeling and simulation, and debugging are some of the skills. Second, this research is limited to a particular topic that was chosen via a need analysis, and it covers 34 biology students and 10 biology teachers. Finally, because of the COVID-19 pandemic’s impact and the SOP that must be followed while doing research in schools, especially when it includes children, this study was done with a limited number of students.

There are multiple areas for further research that stem from this study. Increasing the number of problem-solving computational skills will be helpful to see the impact of ME-CoT implementation on students’ problem-solving skills through computational skills. Besides that, not only metacognitive awareness but students’ interest in learning the specific topic should be focused on to identify whether this ME-CoT module arouses pupils’ interest in learning Biology. Furthermore, the goal of this study is to see the students’ achievement in Biology education, thereby, other topics related to human and animal physiology can be focused on. Finally, the restriction on conducting research in school during the COVID-19 pandemic limited the number of students for this study.

Multiple challenges arose from implementing a curriculum plan to merge STEM education (Biology education) with computer science (computational thinking) to build an interdisciplinary approach. A well-organized learning module with relevant learning goals should be constructed for long-term implementation. Based on the four primary educational theories, this study describes the design and implementation approach for incorporating text-based programming ME-CoT learning Module into current Biology Syllabuses. This study will add some valuable information regarding implementing coding using text-based learning which remains unclear (Papadakis, 2022). We aimed to further experiment to determine the effectiveness of the ME-CoT Learning Module associated with student achievement in Biology, computational thinking, and metacognitive awareness.

## Data Availability Statement

The raw data supporting the conclusions of this article will be made available by the authors, without undue reservation.

## Ethics Statement

The studies involving human participants were reviewed and approved by Ministry of Education, Malaysia. Written informed consent to participate in this study was provided by the participants’ legal guardian/next of kin.

## Author Contributions

NM contributed in the module development, experimentation, and analysis of findings. KO and LH contributed significantly in the theoretical aspect of module development as well discussion of findings and future direction. All authors contributed to the article and approved the submitted version.

## Conflict of Interest

The authors declare that the research was conducted in the absence of any commercial or financial relationships that could be construed as a potential conflict of interest.

## Publisher’s Note

All claims expressed in this article are solely those of the authors and do not necessarily represent those of their affiliated organizations, or those of the publisher, the editors and the reviewers. Any product that may be evaluated in this article, or claim that may be made by its manufacturer, is not guaranteed or endorsed by the publisher.

## References

[B1] Ackermann E. (2001). Piaget’s constructivism, Papert’s constructionism: what’s the difference? *Future Learn. Group Publ.* 5:438. 10.1111/j.1526-4610.2005.t01-1-05013.x 15663618

[B2] Aho A. V. (2012). Computation and computational thinking. *Comput. J.* 55, 832–835. 10.1093/comjnl/bxs074

[B3] Anderton R. S. Evans T. Chivers P. T. (2016). Predicting academic success of health science students for first year anatomy and physiology. *Int. J. High. Educ.* 5, 250–260. 10.5430/ijhe.v5n1p250

[B4] Angeli C. Jaipal-Jamani K. (2018). “Preparing pre-service teachers to promote computational thinking in school classrooms,” in *Computational Thinking in the Stem Disciplines* (Cham: Springer), 127–150.

[B5] Arihasnida A. Nurazmiera M. R. Norhasyimah H. Tamil Selvan S. Siti Nur Kamariah R. (2017). Tahap kemahiran visualisasi bagi mata pelajaran lukisan kejuruteraan di uthm. *J. TVET Practit.* 2 1–8.

[B6] Astuti D. Khasanah U. Romadon S. (2017). *The Effect of Thinking Empowerment by Questioning (TEQ) through Team-Assisted Individualization (TAI) Learning Model to Metacognitive and Learning Achievement.* Cham: Springer.

[B7] Bergan-Roller H. E. Galt N. J. Chizinski C. J. Helikar T. Dauer J. T. (2018). Simulated computational model lesson improves foundational systems thinking skills and conceptual knowledge in biology students. *BioScience* 68 612–621. 10.1093/biosci/biy054

[B8] Bevins S. Price G. (2016). Reconceptualising inquiry in science education. *Int. J. Sci. Educ.* 38, 17–29. 10.1080/09500693.2015.1124300

[B9] Burbaite R. Drasute V. Stuikys V. V. (2018). Integration of computational thinking skills in stem-driven computer science education. *Glob. Eng. Educ. Conf.* 2 1824–1832. 10.1109/EDUCON.2018.8363456

[B10] Çakiroğlu Ü Er B. (2020). Effect of using metacognitive strategies to enhance programming performances. *Inform. Educ.* 19 181–200. 10.15388/INFEDU.2020.09

[B11] Cheah H. M. (2016). Enhancing creative teaching using computational thinking. *Int. Conf. Teach. Learn.* 2016 26–42.

[B12] Chou M. H. (2017). A task-based language teaching approach to developing metacognitive strategies for listening comprehension. *Int. J. Listen.* 31 51–70. 10.1080/10904018.2015.1098542

[B13] Çimer A. (2012). What makes biology learning difficult and effective: students’ views. *Educ. Res. Rev.* 7 61–71. 10.5897/ERR11.205

[B14] Cohen L. Manion L. Morrison K. (2002). *Research Methods in Education.* Abingdon: Routledge.

[B15] del Olmo J. Cózar-Gutiérrez R. González-Calero J. A. del Olmo-Muñoz J. Cózar-Gutiérrez R. González-Calero J. A. (2020). Computational thinking through unplugged activities in early years of Primary Education. *Comput. Educ.* 150:103832. 10.1016/j.compedu.2020.103832

[B16] Falloon G. (2016). An analysis of young students’ thinking when completing basic coding tasks using Scratch Jnr. On the iPad. *J. Comput. Assist. Learn.* 32 576–593. 10.1111/jcal.12155

[B17] Fazilah R. Othman T. Azraai O. (2016). Aplikasi kemahiran proses sains dalam pembelajaran berasaskan masalah untuk matapelajaran biologi. *J. Kurikulum Pengajaran Asia Pasifik* 4 38–46.

[B18] Flavell J. (1979). Metacognition and cognitive monitoring: a new area of cognitive -development inquiry. *Notes Queries* 34 906–911. 10.1093/nq/CLVII.dec14.424-a

[B19] Gagne R. M. (1970). Learning theory, educational media, and individualized instruction. *Learn. Theory* 22.

[B20] Gillott L. Joyce-Gibbons A. Hidson E. (2020). Exploring and comparing computational thinking skills in students who take GCSE Computer Science and those who do not. *Int. J. Comput. Sci. Educ. Sch.* 3 3–22. 10.21585/ijcses.v3i4.77

[B21] Goyal S. Vijay R. S. Monga C. Kalita P. (2016). “Code bits: an inexpensive tangible computational thinking toolkit for K-12 curriculum,” in *TEI 2016 - Proceedings of the 10th Anniversary Conference on Tangible Embedded and Embodied Interaction*, (New York, NY: ACM), 441–447.

[B22] Harrison G. M. Vallin L. M. (2018). Evaluating the metacognitive awareness inventory using empirical factor-structure evidence. *Metacogn. Learn.* 13 15–38. 10.1007/s11409-017-9176-z

[B23] Haslina H. Aliza A. Lye Guet P. Mohd Saferi M. H. (2018). *Using Scratch Programming To Engage Primary School Penggunaan Scratch Programming Untuk Meningkatkan Penglibatan Murid Sekolah Rendah.* Bangi: Universiti Kebangsaan Malaysia, 49–57.

[B24] Hertzog M. A. (2008). Considerations in determining sample size for pilot studies. *Res. Nurs. Health* 31 180–191. 10.1002/nur 18183564

[B25] Higgs L. (2013). Theory in educational research and practice in teacher education. *Bulg. Comp. Educ. Stud.* 11, 105–111.

[B26] Isaac S. Michael W. B. (1995). *Handbook in Research and Evaluation: A Collection of Principles, Methods, and Strategies Useful in the Planning, Design, and Evaluation of Studies in Education and the Behavioral Sciences.* Princeton, NJ: EdITS publishers.

[B27] Jamaludin A. (2008). *Modul dan Pengendalian bimbingan Kelompok.* Serdang: Universiti Putra Malaysia.

[B28] Jamaludin A. (2012). *Modul Motivasi Diri.* Kuala Lumpur: Dewan Bahasa dan Pustaka.

[B29] Kafai Y. B. Burke Q. (2014). *Connected Code: Why Children Need to Learn Programming.* Cambridge, MA: MIT Press.

[B30] Kalelioglu F. Gulbahar Y. Kukul V. (2016). A framework for computational thinking based on a systematic research review. *Baltic J. Modern Comput.* 4 583–596.

[B31] Kamisah O. (2022). *Contextualizing Computational Thinking Disposition Framework From an Affective Perspective.* Bangi: Universiti Kebangsaan Malaysia, 390–412.

[B32] Karimah M. Noraidah S. A. Tengku Siti Meriam T. Noorazean M. A. (2021). Validation of the components and elements of computational thinking for teaching and learning programming using the fuzzy delphi method. *Int. J. Adv. Comput. Sci. Appl.* 12 80–88. 10.14569/IJACSA.2021.0120111

[B33] Karimah M. Noraidah Sahari A. Tengku Siti Meriam W. T. Noorazean M. (2020). Analysis of the requirements of computational thinking skills to overcome the difficulties in learning programming. *Int. J. Adv. Comput. Sci. Appl.* 11 244–253. 10.14569/ijacsa.2020.0110329

[B34] Kaufmann O. T. Stenseth B. (2021). Programming in mathematics education. *Int. J. Math. Educ. Sci. Technol.* 52 1029–1048. 10.1080/0020739X.2020.1736349

[B35] Kazimoglu C. Kiernan M. Bacon L. MacKinnon L. (2012). Learning programming at the computational thinking level via digital game-play. *Proc. Comput. Sci.* 9 522–531. 10.1016/j.procs.2012.04.056

[B36] Koc I. Kuvac M. (2016). Preservice science teachers’ metacognitive awareness levels. *Eur. J. Educ. Stud.* 0 43–63.

[B37] Kotsopoulos D. Floyd L. Khan S. Namukasa I. K. Somanath S. Weber J. (2017). A pedagogical framework for computational thinking. *Digital Exp. Math. Educ.* 3 154–171. 10.1007/s40751-017-0031-2

[B38] Kozulin A. (2015). Vygotsky’s theory of cognitive development. *Int. Encyclopedia Soc. Behav. Sci.* 25 322–328. 10.1016/j.nepr.2019.03.010 30884419

[B39] KPM (2016). *Buku Penerangan Kurikulum Standard Sekolah Menengah (KSSM).* Putrajaya: Bahagian Pembangunan Kurikulum.

[B40] KPM (2017). *Laporan Kebangsaan TIMSS 2015.* Putrajaya: KPM, 1–112.

[B41] Krathwohl D. R. (2002). A revision of Bloom’s taxonomy an overview. *Am. J. Psychol.* 41 219–225. 10.1207/s15430421tip4104

[B42] Krpan D. Mladenovic S. Zaharija G. (2017). “Mediated transfer from visual to high-level programming language,” in *Proceedings of the 2017 40th International Convention on Information and Communication Technology, Electronics and Microelectronics, MIPRO 2017*, (Rijeka: MIPRO), 800–805.

[B43] Kusuma M. D. Rosidin U. Abdurrahman A. Suyatna A. (2017). The development of higher order thinking skill (Hots) instrument assessment in physics Study. *IOSR J. Res. Method Educ.* 07 26–32. 10.9790/7388-0701052632

[B44] Kyairaniah A. H. Mohd Isa H. Lubis M. A. (2017). Tahap kemahiran metakognitif murid sekolah menengah di kawasan felda dalam pembelajaran pendidikan Islam. *ASEAN Comp. Educ. Res. J. Islam Civil.* 1 94–106.

[B45] Lay Ah Nam Kamisah O. (2017). Developing 21st century skills through a constructivist-constructionist learning environment. *STEM Educ.* 3 205–216.

[B46] Li Y. Schoenfeld A. H. diSessa A. A. Graesser A. C. Benson L. C. English L. D. (2020). Computational thinking is more about thinking than computing. *J. STEM Educ. Res.* 3 1–18. 10.1007/s41979-020-00030-2 32838129 PMC7234448

[B47] Lin Y. Weintrop D. (2021). The landscape of Block-based programming: characteristics of block-based environments and how they support the transition to text-based programming. *J. Comput. Lang.* 67:101075. 10.1016/j.cola.2021.101075

[B48] Lynne C. (2008). PS1 pilot studies. *Medsurg Nurs.* 17:411. 19248407

[B49] Maharani S. Kholid M. N. Pradana L. N. Nusantara T. (2019). Problem solving in the context of computational thinking. *Infinity J.* 8:109. 10.22460/infinity.v8i2.p109-116

[B50] Mazli Sham A. Saemah R. (2014). Gaya pembelajaran dan kesedaran metakognitif dalam kalangan pelajar aliran sains. *Proc. Sosial Sci. Res.* 12 712–719.

[B51] Mohamad Masrizan A. P. (2019). Exploring metacognition regulation among biology student at labuan matriculation college mohamad. *J. Kurikulum* 53 1–16.

[B52] Mohd Noor A. (2012). *Redesigning Classroom Pedagogy.* Singapore: National Institute of Education Singapore.

[B53] Mohd Sidek N. Jamaludin A. (2005). *Pembinaan Modul: Bagaimana Membina Modul Latihan Dan Modul Akademik.* Serdang: Universiti Putra Malaysia.

[B54] Odom L. R. Morrow J. R. (2006). What’s this r? A correlational approach to explaining validity, reliability, and objectivity coefficients. *Meas. Phys. Educ. Exerc. Sci.* 10 137–145. 10.1207/s15327841mpee1002_5 33486653

[B55] Oluk A. Korkmaz Ö (2016). Comparing students’ scratch skills with their computational thinking skills in terms of different variables. *Int. J. Modern Educ. Comput. Sci.* 8 1–7. 10.5815/ijmecs.2016.11.01

[B56] Otterborn A. Schönborn K. J. Hultén M. (2020). Investigating preschool educators’ implementation of computer programming in their teaching practice. *Early Childhood Educ. J.* 48 253–262. 10.1007/s10643-019-00976-y

[B57] Papadakis S. (2022). Can preschoolers learn computational thinking and coding skills with scratchjr? A systematic literature review. *Int. J. Educ. Reform* 2022:10567879221076077.

[B58] Papadakis S. Kalogiannakis M. (2019). “Evaluating a course for teaching advanced programming concepts with scratch to preservice kindergarten teachers: a case study in Greece,” in *Early Childhood Education*, eds Farland-Smith D. (Hoboken, NJ: Wiley).

[B59] Papert S. (1996). An exploration in the space of mathematics educations. *Int. J. Comput. Math. Learn.* 1, 95–123. 10.1007/BF00191473

[B60] Pedaste M. Mäeots M. Siiman L. A. de Jong T. van Riesen S. A. N. Kamp E. T. (2015). Phases of inquiry-based learning: definitions and the inquiry cycle. *Educ. Res. Rev.* 14 47–61. 10.1016/j.edurev.2015.02.003

[B61] Periya S. N. Moro C. (2019). Applied learning of anatomy and physiology: virtual dissectiontables within medical and health sciences education. *Bangkok Med. J.* 15 121–127. 10.31524/bkkmedj.2019.02.021

[B62] Piaget J. (1972a). Intellectual evolution from adolescence to adulthood. *Hum. Dev.* 15 1–12. 10.1159/000271225

[B63] Piaget J. (1972b). The role of imitation in the development of representational thought. *Int. J. Ment. Health* 1 67–74. 10.1080/00207411.1972.11448598

[B64] Ping I. L. L. Halim L. Osman K. (2020). Explicit teaching of scientific argumentation as an approach to developing argumentation skills, science process skills, and biology understanding. *J. Baltic Sci. Educ.* 19 276–288. 10.33225/jbse/20.19.276

[B65] Pressley F. D. MacKinnon G. Waller T. G. (1985). *Metacognition, Cognition, and Human Performance.* Cambridge, MA: Academic Press.

[B66] Puganesri K. Puteh S. (2019). Computer science education in Malaysia schools: the challenges of enhancing computational thinking skills. *Int. J. Eng. Adv. Technol.* 8 441–444. 10.35940/ijeat.F1080.0986S319

[B67] Ragbir Kaur Joginder Singh (2011). *Panduan Ilmu Pendidikan Komprehensif unutk KPLI(Sekolah Rendah).* Selangor: KUMPULAN BUDIMAN SDN.

[B68] Ramlan M. Ghazali D. (2018). *Aplikasi Kaedah Fuzzy Delphi Penyelidikan Sains Sosial.* Kuala Lumpur: Penerbit Universiti Malaya.

[B69] Reinoso Tapia R. Delgado-Iglesias J. Fernández I. (2019). Learning difficulties, alternative conceptions, and misconceptions of student teachers about respiratory physiology. *Int. J. Sci. Educ.* 41 2602–2625. 10.1080/09500693.2019.1690177

[B70] Román-González M. Moreno-León J. Robles G. (2017). Complementary tools for computational thinking assessment. *Proc. Int. Conf. Comput. Think. Educ.* 17 154–159.

[B71] Rubinstein A. Chor B. (2014). Computational thinking in life science education. *PLoS Comput. Biol.* 10:e1003897. 10.1371/journal.pcbi.1003897 25411839 PMC4238948

[B72] Samri C. Osman K. Nayan N. A. (2020). Level of computational thinking skills among secondary. *Sci. Educ. Int.* 31 159–163. 10.33828/sei.v31.i2.4

[B73] Schraw G. Dennison R. S. (1994). Assessing metacognitive awareness. *Contemp. Educ. Psychol.* 19 460–475. 10.1006/ceps.1994.1033

[B74] Stripeikaitė I. (2017). “Skipping the baby steps”: the importance of teaching practical programming before programming theory. *Lecture Notes Comput. Sci.* 22 319–330. 10.1007/978-3-319-70111-0_30

[B75] Sun L. (2013). The effect of metacognitive learning strategies on English learning. *Theory Pract. Lang. Stud.* 3 2004–2009. 10.4304/tpls.3.11.2004-2009

[B76] Susan S. Nurfaradilla M. N. (2019). Teachers’ concern towards applying computational thinking skills in teaching and learning. *Int. J. Acad. Res. Bus. Soc. Sci.* 9 296–310. 10.6007/ijarbss/v9-i1/5398

[B77] Tsarava K. Leifheit L. Ninaus M. Román-González M. Butz M. V. Golle J. (2019). “Cognitive correlates of computational thinking,” in *Proceedings of the 14th Workshop in Primary and Secondary Computing Education*, Glasgow, 1–9.

[B78] Vygotsky L. S. (1979a). Consciousness as a problem in the psychology of behavior. *Sov. Psychol.* 17, 3–35. 10.2753/rpo1061-040517043

[B79] Vygotsky L. S. (1979b). The development of higher forms of attention in childhood. *Sov. Psychol.* 18, 67–115. 10.2753/rpo1061-0405180167

[B80] Wing J. (2011). *Research Notebook: Computational Thinking—What and Why? The Link Magazine.* Available online at: http://www.cs.cmu.edu/link/research-notebook-computational-thinking-what-and-why (accessed June 23, 2015).

[B81] Wing J. M. (2006). Computational thinking. *Concurrences* 49 33–35.

[B82] Yağcı M. (2019). A valid and reliable tool for examining computational thinking skills. *Educ. Inf. Technol.* 24, 929–951. 10.1007/s10639-018-9801-8

[B83] Zapata-Caceres M. Martin-Barroso E. Roman-Gonzalez M. (2020). “Computational thinking test for beginners: design and content validation,” in *Proceedings of the IEEE Global Engineering Education Conference, EDUCON, 2020-April*, (Piscataway, NJ: IEEE), 1905–1914.

